# Enteroendocrine Dynamics – New Tools Reveal Hormonal Plasticity in the Gut

**DOI:** 10.1210/endrev/bnaa018

**Published:** 2020-06-12

**Authors:** Joep Beumer, Helmuth Gehart, Hans Clevers

**Affiliations:** 1 Hubrecht Institute, Royal Netherlands Academy of Arts and Sciences (KNAW) and UMC Utrecht, CT Utrecht, The Netherlands; 2 Institute for Molecular Health Sciences, ETH Zurich, Zurich, Switzerland; 3 Oncode Institute, Hubrecht Institute, CT Utrecht, The Netherlands

**Keywords:** enteroendocrine, intestine, plasticity, differentiation, model systems, organoid

## Abstract

The recent intersection of enteroendocrine cell biology with single-cell technologies and novel in vitro model systems has generated a tremendous amount of new data. Here we highlight these recent developments and explore how these findings contribute to the understanding of endocrine lineages in the gut. In particular, the concept of hormonal plasticity, the ability of endocrine cells to produce different hormones over the course of their lifetime, challenges the classic notion of cell types. Enteroendocrine cells travel in the course of their life through different signaling environments that directly influence their hormonal repertoire. In this context, we examine how enteroendocrine cell fate is determined and modulated by signaling molecules such as bone morphogenetic proteins (BMPs) or location along the gastrointestinal tract. We analyze advantages and disadvantages of novel in vitro tools, adult stem cell or iPS-derived intestinal organoids, that have been crucial for recent findings on enteroendocrine development and plasticity. Finally, we illuminate the future perspectives of the field and discuss how understanding enteroendocrine plasticity can lead to new therapeutic approaches.

## Graphical Abstract

**Graphical Abstract:**
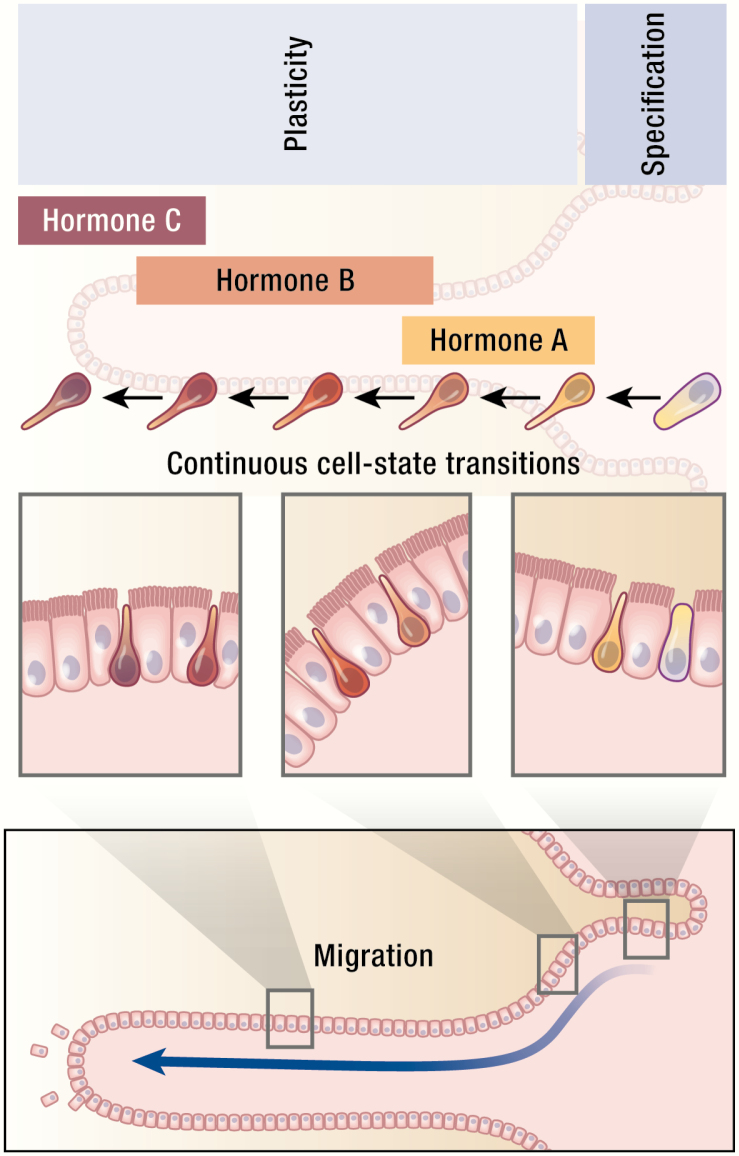

**Essential Points**

- Enteroendocrine cells are rare hormone-producing cells in the gut that control key physiological processes related to food intake by secreting more than 20 different peptides.
- Types of secreted hormones change over the lifetime of an individual enteroendocrine cell.
- The hormonal repertoire of an individual cell is limited by enteroendocrine lineage (5 lineages total) and gastrointestinal region–specific epigenetic landscape.
- Hormone expression within these limits is dynamically controlled by the action of changing morphogen signaling during the migration of cells from crypt to villus.
- Adult and pluripotent–stem-cell–derived organoids represent emerging model systems to assess development and function of enteroendocrine cells.

The intestinal epithelium is generally associated with nutrient uptake and barrier function. However, it is also the largest endocrine organ in our body. In an average human, around 100 million intestinal endocrine cells, called enteroendocrine cells (EECs), are shed every day and regenerated by newly differentiating cells. Despite the impressive number, EECs only constitute 1% of the intestinal epithelium. EECs are scattered throughout the epithelium and produce more than 20 intestinal hormones. Which hormones are produced in an individual cell and which stimulus causes the release depends on the EEC type. Classically EECs are distinguished by the main hormone they secrete: enterochromaffin (EC) cells (serotonin, 5-HT), I cells (cholecystokinin, CCK), K cells (gastric inhibitory peptide, GIP), L cells (glucagon-like peptide 1, GLP-1), X cells (ghrelin, GHRL), S cells (secretin, SCT), D cells (somatostatin, SST), and N cells (neurotensin, NTS) were originally described. However, reports of multihormonal cells testify to the insufficiency of the single letter system (1-3). Alternative classification systems describing region, species, and detected hormones (e.g., J_M_^GIP+SST+GCG-PYY-^ for a mouse jejunal EEC) (4) avoid putting cells in oversimplified “boxes,” but are difficult to use in practice.

Intestinal hormones serve a wide range of functions. Postprandially released hormones (peptide YY [PYY], 5-HT, CCK, GLP-1, GIP) or hormones released during fasting (GHRL, 5-HT) have direct influence on glucose homeostasis. GLP-1 and GIP, called incretins, potentiate insulin release from the beta cells in the pancreas (5). In contrast, GHRL release has the opposite effect (6). Intestinal hormones act not only indirectly via the pancreas on blood glucose levels, but also by controlling the flow of nutrients through the intestine. CCK, GLP-1, and PYY, for example, delay gastric emptying once nutrients have reached the distal small intestine (7). Finally, gut hormones control metabolic adaptation by modulating appetite. GHRL is released in anticipation of a meal, whereas postprandially released GLP-1, CCK, and PYY induce satiety in the central nervous system (8, 9). Although less established than their metabolic roles, EECs have also been linked to orchestrating mucosal immunity (10, 11). Since EECs express receptors for microbial metabolites, secrete cytokines upon stimulation, and produce hormones that can act directly on immune cells, they are equipped to act as sentinels and coordinators of intestinal immunity. However, the extent of this role has yet to be mechanistically assessed.

Until recently it has been unclear whether the observed diversity in EECs (more than 20 reported cell types) is a product of an equally high number of independent differentiation pathways from the common enteroendocrine progenitor stage. However, advances in endocrine biology, single-cell sequencing, and organoid technology have partially reconciled the original classification with the observed functional diversity by introducing the concept of hormonal plasticity: the ability of a single EEC to go through various functional states with differing hormonal repertoire. In this review, we discuss these recent findings and try to synthetize a comprehensive picture of the signals and factors that determine and modulate enteroendocrine fate. Finally, we will highlight the advantages and disadvantages of the enteroendocrine in vitro systems that made these findings possible and discuss the future perspectives of the field.

## Enteroendocrine Cell Fate Determination

Until the 1960s, the substantial resemblance of EECs to neurons (e.g., the production of neurotransmitters and synapse-like projections) led to a general belief that endocrine epithelial cells stem from neural crest cells migrating into the gut epithelium (12). The chromaffin cells of the adrenal medulla, named after their characteristic stain with chrome salts resembling intestinal ECs, are similarly known to derive from the neural crest. The first embryonic lineage-tracing techniques refuted this model later. When quail mesoderm was combined with chick endoderm during embryonic development, all of the resulting EEC cells were of chick origin (13). Thus, it was definitively shown that all EECs are of endodermal origin.

**Figure 1.**
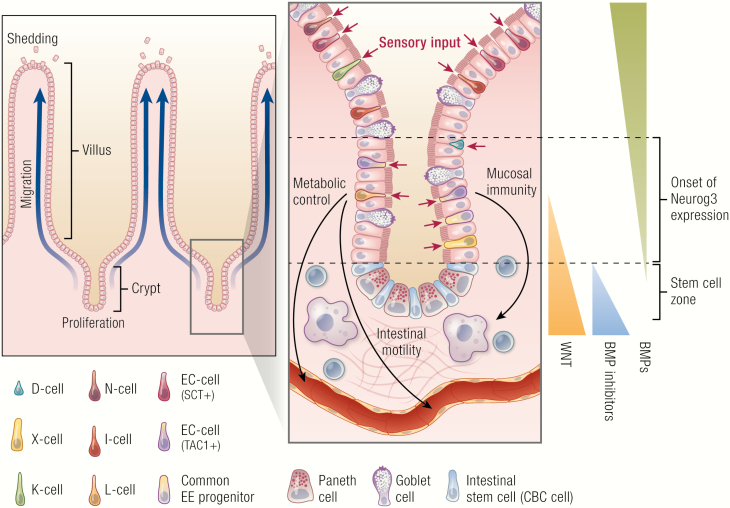
Subtypes and functions of enteroendocrine cells. All cells of the intestinal epithelium including enteroendocrine cells (EECs) are generated in the stem-cell zone by continuously proliferating crypt-base columnar (CBC) intestinal stem cells. Once ejected from the stem-cell zone, daughter cells begin to differentiate into one of the many intestinal cell types and migrate towards the villus, where cells are eventually shed. EECs start their differentiation when cells outside the stem-cell zone lose Notch activity and upregulate the pro-endocrine transcription factor neurogenin-3 (Neurog3). Initiation of Neurog3 expression can occur in a wide range of the crypt epithelium, with different exposure levels to the morphogens WNT and BMP. This difference in environmental signals may have direct influence on lineage specification of individual EECs. Although, EECs make up less than 1% of the epithelium, they secrete about 20 different products. Subtypes are identified by a single-letter code based on their principal hormone. Sensory input from the intestinal lumen controls secretion of these hormones that regulate metabolism, mucosal immunity and intestinal motility.

In an attempt to establish a common precursor to the differentiated cells of the intestinal epithelium, Cheng and Leblond performed radioactive nucleotide labeling and followed the trace over time (14). This work identified crypt base columnar (CBC) cells, slender cells in between Paneth cells (Fig. 1), which passed on their radioactive label to all differentiated lineages including EECs (14). Final proof for the stem-cell nature of CBCs was only provided in 2007, when the G-protein–coupled receptor *Lgr5* was identified as a marker of CBCs. Lineage tracing based on *Lgr5* expression demonstrated the ability of CBCs to regenerate the whole intestinal epithelium and themselves throughout lifetime (15). Single Lgr5^+^ CBC cells can be isolated and generate three-dimensional (3D) in vitro organoid cultures (discussed later) containing all intestinal cell types including EECs (16). Thus, like the rest of the intestinal epithelium, EECs are continuously generated by CBCs at the bottom of the crypt and are shed into the intestinal lumen at the end of their lifespan (days to weeks).

### Mechanisms of EEC specification

The intestinal epithelium displays a rapid turnover of 4 to 5 days, which is highly atypical for an endocrine organ. For example, the endocrine pancreas is largely generated during embryonic development and shows little turnover in adult homeostasis (17). Intestinal stem cells, on the other hand, divide continuously at the bottom of crypts, while their differentiating offspring move in a conveyor belt-like motion towards the villus tips, where cells are shed (Fig. 1). Two main branches of differentiated cell types are generated: the absorptive enterocytes and the secretory cells, including mucus-secreting goblet cells, antimicrobial and stem cell niche-factor- (epidermal growth factor [EGF], WNT, Notch-ligand) producing Paneth cells, and a range of different EECs. A binary switch controlled by Notch signaling maintains a steady balance between secretory and absorptive cells via lateral inhibition. Cells that do not receive Notch signals when leaving the stem-cell zone acquire a secretory fate. These secretory progenitors will upregulate Notch ligands and induce Notch activation in all surrounding cells that consequently differentiate to the absorptive fate.

Active Notch signaling stimulates expression of Hairy/enhancer of split 1 (Hes1) (18, 19), a potent repressor for the basic helix-loop-helix (bHLH) transcription factors Atoh1 (also known as Math1) and neurogenin-3 (Neurog3). Whereas the former is important for the production of all secretory cells, the latter is the key regulator for EEC cell formation. Mice deficient for Neurog3 completely lack all EEC subtypes in the small and large intestine. Conversely, transgenic overexpression greatly increases the generation of all EEC lineages (20, 21). In addition to transcriptional regulation, Neurog3 in the endocrine pancreas is also post-transcriptionally regulated by several cyclin-dependent kinases that phosphorylate Neurog3 and cause its proteasomal degradation (22, 23). These findings indicate that dividing progenitor cells actively degrade Neurog3. On the other hand, Neurog3 actively promotes cell cycle exit by stimulating expression of the cell cycle inhibitor *Cdkn1a* (24). A study by the Winton lab followed the fate of particularly slow dividing cells in the intestine and found that these turned indeed into Paneth cells and EECs, but never into Goblet cells (25). Along the same line, we have found that cell cycle inhibition of intestinal stem cells predispose these to an EEC fate (26). These findings corroborate the earlier mechanistic studies and indicate that active proliferation in secretory progenitors favors goblet cell differentiation, whereas Neurog3 activity and thus endocrine fate specification is limited to progenitors with low division rates.

Another player recently implicated in the regulation of EEC differentiation is mechanical force. EEC progenitors in the gut of fruit flies express the mechano-sensor *Piezo*, a membrane-spanning protein that functions as an ion channel responding to forces (27). Stretch forces on the intestinal epithelium cause an increase in cytosolic Ca^2+^ levels specifically in EEC progenitors, which stimulates their differentiation (27). Sensitivity to mechanical force is also conserved in mammalian EEC generation. Hippo signaling, a mechanically regulated pathway in mammals, has been implicated in the regulation of secretory cell differentiation (28). When cellular density is low, Hippo signaling is turned off and YAP translocates to the nucleus to activate target gene expression. Differences in Hippo activation, potentially due to uneven levels of mechanical stress, appear to be a main driver in symmetry breaking between intestinal stem cells and their secretory offspring (29). This is why, artificial continuous activation or inactivation of YAP block the development of secretory cells, including EECs (30).

Although the niche signals driving segregation of EECs from absorptive enterocytes (Notch), Paneth cells (Wnt) and Goblet cells (cell cycle, EGF, mechanical forces) are becoming elucidated (26, 31), the downstream factors causing the different EEC subtypes to be produced are largely unknown.

Recent advances in the field of single-cell sequencing have enabled researchers to study fate determination of EECs on a single-cell level. An intestinal cell atlas by Haber et al described the diversity of intestinal endocrine cells in the crypt and defined previously unknown endocrine cell populations (1). Building on this first single-cell dataset, our own work reconstructed the complete developmental trajectory and all transcriptional event in the course the differentiation of all EEC subtypes on single-cell level with real-time resolution. To do so, we created a time-resolved Neurog3 reporter mouse (Neurog3Chrono), which combined high temporal resolution with the ability to report even the lowest levels of Neurog3 expression (32). The Neurog3Chrono system showed that cell position within the crypt at the onset of Neurog3 expression was not uniform, but distributed across a broad region along the crypt (Fig. 1). Thus, it is tempting to speculate that the position along the signaling gradients within the crypt plays an important role in determining the final lineage of the differentiating cell. By combining the Neurog3Chrono system with single-cell sequencing and functional studies in organoids we were able to determine that there were only 5 distinct lineages of EECs. However, these 5 lineages could generate cells with highly divergent hormonal profiles. This was possible, since many presumably mature EEC types (e.g., L-cells) were transition stages in the lifetime of an individual cell that switched its hormonal repertoire as it moved through signaling gradients along the crypt-villus axis.

In summary, an EEC undergoes 3 stages of differentiation from CBC to hormone-secreting cell. During fate determination, secretory fate is induced in cells outside the stem-cell zone at the bottom of the crypt by the absence of Notch activation. This is followed by lineage specification, when Neurog3 activity in non-cycling cells induces 1 of 5 individual differentiation programs. This specification step determines the hormonal repertoire of each individual EEC. In the final stage of hormone plasticity, signaling gradients along the crypt-villus axis dynamically control which hormone within the repertoire is being actively produced. This allows a single cell to produce different hormones at various timepoints throughout its life. The significant body of evidence that supports this dynamic versus the older static model of differentiation is the subject of the next section.

### Hormone plasticity

EECs were usually described as hormone factories with static peptide repertoires, often only a single hormone. This led to the single letter-coding of the EEC subtypes described above. With the advent of more sensitive imaging techniques or transcriptomic measurements, a more complex set of combinations of hormones was identified (1, 33). It remained unknown, however, if each observed hormone combination was generated by an independent cell lineage, or whether it was sign of changing hormonal profiles in individual cells.

The first evidence for the latter appeared in the 1980s. Radioactive thymidine labeling showed rapid labeling of serotonin-producing cells, while secretin-producing cells were labeled only after 2 days and were restricted to the villus (34, 35). This led the authors to conclude that the differentiation of secretin-producing cells does not happen before these reach the villus. The Gordon lab in the early 1990s more widely characterized crypt-villus heterogeneity of EEC products and for the first time suggested possible lineage relationships between EECs making different hormones in these areas. This extensive immunohistochemical work identified TAC1 and serotonin co-expression in the intestinal crypt, while in the villus tips serotonin overlapped with secretin (36). In the villus bottom, rare TAC1, serotonin, and secretin triple-positive EECs could be found, suggesting a lineage of EECs undergoing transitions in the produced peptides (36). Subsequent work combined hormone staining with BrdU injections to confirm that TAC1+ cells were rapidly generated from dividing progenitors, while secretin+ cells did not appear until 2 days later (37). Similarly, GLP-1 was recognized as a typical crypt hormone that follows similar temporal and spatial patterns as TAC1 (38). Functional evidence that individual EECs can dynamically produce different hormone peptides appeared 2 decades later: Exploiting a diphtheria-toxin receptor driven by the pre-proglucagon promoter (*Gcg*, precursor for Glp-1), the Schwartz lab specifically ablated L-cells defined by *Gcg* expression and followed the loss and reappearance of different hormones (39). Within 24 hours, the vast majority of GLP-1^+^ cells was lost, while also a significant but smaller proportion of NTS and PYY positive cells disappeared. GLP-1^+^ cells reappeared much quicker than PYY- and NTS-producing cells (39), which suggests that the former may give rise to the latter. The Neurog3Chrono model further enabled researchers to follow individual EECs over their lifetime, and showed that L-cells indeed upregulate *Pyy* and *Nts* after downregulating *Gcg*, 3 days after the initiation of Neurog3-expression (32). The model demonstrated a similar temporal transition for Tac1, serotonin and secretin. When TAC1^+^ cells were lineage-traced in the intestinal epithelium, all EECs that produced serotonin and secretin in the villus were labeled, even though most stained negative for TAC1 (40). This same tracing experiment yielded no labeling of the other EEC lineages, highlighting the committed nature of EEC subtypes and the inherent limits of plasticity (40).

**Figure 2.**
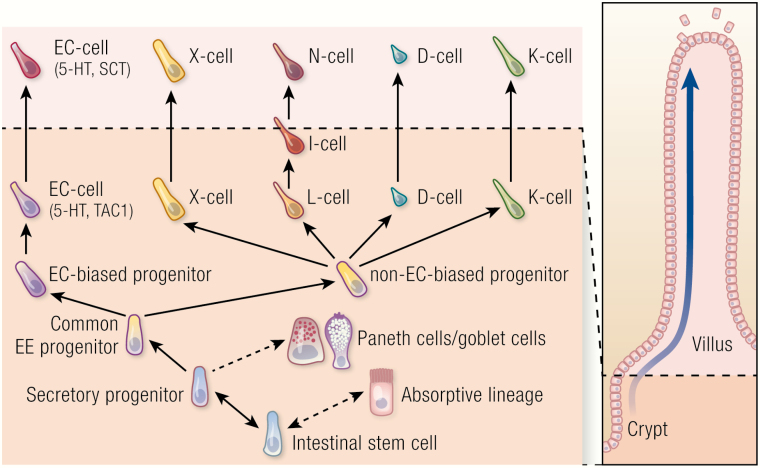
Hormone plasticity among enteroendocrine cells (EECs). Intestinal stem cells generate absorptive enterocytes and all secretory cell types, including EECs, Paneth cells, and goblet cells. All EECs share a common, Neurog3-positive progenitor cell. Enterochromaffin cells (ECs) later derive from a separate, EC-biased progenitor, while the other hormone-producing cells share a common non-EC biased progenitor. During migration along the crypt-villus axis, EECs switch their hormone repertoire within the limits of the 5 individual EEC lineages in response to BMP exposure. BMP signals, which increase the further the cells move away from the crypt, repress Tac1 in ECs, while inducing secretin (Sct). Likewise, BMP represses Glp-1, but induces expression of Nts. This hormone switch changes the single-letter classification of an individual L-cell as it migrates to the villus tip, to I-cell and finally N-cell.

Different lines of evidence suggest that hormone-switching should involve signals unique to the crypt or villus such as morphogens or luminal components, rather than a cell-intrinsic trans-differentiation pathway of the EEC subset. Bjerkness and Cheng described a population of EECs that did not follow the collective stream of cells from the crypt to the villus tip but stayed in the crypt after their generation (41). This was a first indication that EECs have the ability to remain stationary within a moving epithelial cell sheet. Interestingly, these cells failed to express villus hormones such as secretin, and maintained expression of the crypt-restricted Tac1 or Glp-1 as long as they remained in the crypt (37, 38), which suggested a link between EEC localization and hormone expression. Using the Neurog3Chrono system, we could show that all EECs stayed in the crypt during EEC lineage specification (approximately first 48 hours after EEC fate commitment) and thus did not move immediately towards the villus with the epithelial cell sheet. This stands in stark contrast to absorptive cells of the intestinal epithelium, which would have reached the middle of the villus by that time. Once the maturing EECs had gained the ability to produce and secrete hormones (40-60 hours after lineage commitment, depending on lineage), the majority of EECs started to move up the crypt villus-axis, while a smaller population remained in the crypt for longer periods (32). The observed hormone switching of L- and EC-cells coincided with movement of these populations from the crypt to the villus (Fig. 2). This suggests that the differing environments in crypt and villus could indeed drive hormone switching. Further mechanistic insight into this process came from organoid experiments. EECs in vitro in murine intestinal organoids displayed typical expression profiles of hormones found in the crypt, while lacking high expression of villus hormones. For example, ECs always co-expressed Tac1 and Tph1 (tryptophan hydroxylase 1, the rate-limiting enzyme for serotonin production) and displayed low secretin levels (26, 40, 42). Multiple morphogen gradients exist in the intestinal epithelium along the crypt-villus axis, including WNT, bone morphogenetic protein (BMP), EGF, and hedgehog signals that are not naturally established in organoids (43). By modulating these pathways in organoids, we identified BMP signaling as one of the main regulators of hormone plasticity (40). BMP signals, which are high in the villus compartment, are deliberately inhibited in organoid cultures to prevent excessive differentiation of stem cells and to allow their expansion. When BMP signaling is activated in organoids, L-cells lose Glp-1 and increase secretin, neurotensin and Pyy expression. ECs similarly gain secretin, while Tac1 expression is halted (40). Thus, the organoid switches from a crypt to a villus state. Inhibition of BMP signaling in vivo extends the expression of hormones normally restricted to the crypt into the villus region, while repressing the expression of the typical villus hormones (40). This makes the BMP-signaling pathway an interesting factor in targeted manipulation of EEC composition. For example, intestinal BMP inhibition in patients with type 2 diabetes could significantly boost the numbers of Glp-1–producing cells and thus blood glucose control.

Plasticity in EECs could even go beyond hormone switching. Recent studies have shown that EECs, similar to early progenitor cells destined towards enterocytes or secretory lineages (44, 45), have the ability to reacquire intestinal stem cell state upon damage (46, 47). Indeed, the nonproliferative nature of EECs and their close proximity to the stem-cell zone, makes EECs an ideal reserve stem-cell population, when highly proliferative cells have been wiped out by chemotherapy or irradiation.

In summary, the uneven distribution of hormones such as Glp-1 or secretin between crypt and villus has long indicated that position along the crypt-villus axis directly influences EEC cell states. However, only recently, with the advent of organoid models, improved imaging techniques, and single-cell transcriptomics, could researchers demonstrate that specific signals, such as changing BMP abundance, guide an individual EEC through states with distinctively different hormonal profiles. This heterogeneity along the crypt-villus axis is further exacerbated by the variability in tissue composition from the proximal to the distal intestine.

### Heterogeneity along the intestinal tract

The majority of EEC subtypes are not found in constant ratios across the gastrointestinal tract. The enterochromaffin cells and D-cells are the exception to this rule. L-cells are abundant in the distal small intestine and almost absent in the duodenum, while GIP and K-cells display the reverse trend (48). The underlying mechanisms for these differential hormone programs are not yet fully understood. However, potential explanations can be derived from organoid cultures, which have been found to maintain their location-specific function in vitro (49). For example, the bile acid transporter *Slc10a2* is unique to the ileum, and was accordingly only expressed in ileal organoids. Likewise, the EEC composition of mouse and human intestinal organoids mimics the regional identity of the tissue of origin even during long-term culture (26, 40). Since intestinal organoids grow as pure epithelium in a defined medium, nonepithelial niche factors or nutrients can be excluded as potential regulators of regional differences. This leaves differing DNA methylation patterns and epigenetic imprinting that is established during embryonic development and maintained thereafter as likely explanation. Indeed, organoid cultures displayed identical DNA methylation patterns compared with their source tissue (50). When methylation patterns were disrupted using DNA methyltransferase inhibitors in organoids, ectopic expression of multiple regionally restricted markers occurred (50). Although it has not yet been demonstrated, it is likely that the same treatment would reset the enteroendocrine repertoire of organoids derived from different intestinal regions.

In summary, EEC plasticity acts within strict limits imposed by initial lineage specification and regional identity. Lineage specification creates 5 distinct cell types of EECs that do not interconvert, but have the capacity to express a certain cell type–specific set of hormones. Which hormones among this set are expressed in an individual EEC is determined statically by regionally defined epigenetic landscape (e.g., proximal or distal) and dynamically by different environmental cues along the crypt-villus axis, such as BMP signal intensity. Thus, many EEC cell types that have been defined in the past (e.g., L-cells) do not describe the static identity of an EEC, but rather a transient state that can be acquired or lost by a specific EEC lineage depending on environmental cues.

## Model Systems for the Study of EEC Biology

Multiple model systems have been employed to aid the study of EECs, including hormone reporters to purify populations or follow the effects of genetic or chemical challenges on subsets of EECs. Mouse models have made invaluable contributions to our understanding of the enteroendocrine system. However, mechanistic understanding of enteroendocrine cell fate and function necessitates representative in vitro systems that are accessible for functional testing and easily accessible for manipulation such as genome editing.

### Cell lines

Cell lines are immortalized by alterations circumventing senescence, such as the introduction of the SV40 large-T antigen, and have been generated from a wide variety of animals. For example, the murine GLUTag cell line was generated from a mouse endocrine tumor, and has been widely used as a model for regulation of GLP-1 secretion (51). The BON cell line is another commonly used model for EEC function that produces serotonin and is derived from a metastatic human carcinoid tumor (52). Although these models have been instrumental for early insights into secretion mechanisms, these cells do not capture the complex features underpinning EEC development and behavior. Cell lines display high proliferation rates, which makes them easy to expand, but also renders them inherently nonrepresentative for the antiproliferative state that is necessary for EEC differentiation in vivo (53). Moreover, cellular interactions between different cell types of the intestinal epithelium are completely absent due to the homogeneous nature of cell lines. These shortcomings made the development of more complex, but also more representative in vitro systems necessary.

### Adult stem cell-derived organoids

**Figure 3.**
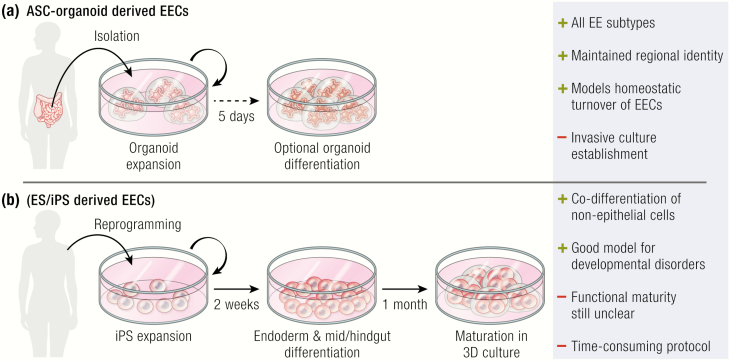
Organoid models for the study of enteroendocrine cells (EECs). A) Adult stem cell (ASC)-derived organoids are established from mature tissue, and reflect the region of origin. Thus, only the constituent cell types including EECs of that region of the intestinal tract are generated in culture. Differentiation protocols are generally simple, requiring few growth-factor changes and short time windows to obtain mature cells. All EEC subtypes can be generated. B) Embryonic or induced pluripotent stem cells can readily be derived from any adult cell type, and do not require invasive surgeries from the gastrointestinal tract. To generate adult intestine, developmental principles are mimicked that occur during embryonic development. Other cell types that exist alongside the epithelium, such as mesenchyme and enteric neurons, can simultaneously be induced or added, allowing for studying the interaction between these cells and EECs.

In the last decade, organoids or “mini-guts” have evolved as alternative to the use of cell lines. Organoids are 3D cultures that can be grown from a single adult mouse or human stem cell (ASC) and expand indefinitely in a gel-matrix (Fig. 3). These cells are grown in defined cocktail of growth factors mimicking the stem-cell niche. Mouse small intestinal organoids contain all different intestinal epithelial cell types at near-normal ratios, and are therefore excellent models to study interaction among EECs and with other epithelial cell types (16). These organoids can be exposed to different signaling pathway modulators to enrich EECs. For example, the inhibition of WNT, Notch and EGFR/MEK boosted the differentiation of EECs in mouse small intestinal organoids from less than 1% to almost 50% (26). These cultures have been valuable to identify developmental regulators of EECs by modulation of certain niche signals—such as BMP signaling—or by loss-of-function experiments using CRISPR-Cas9–mediated knockout (32, 40). The specific luminal signals that dictate hormone secretion are difficult to distinguish in mice due to the complexity of the diet and microbiome. Organoids allow for a controlled exposure to individual metabolic molecules. Such experiments are essential to untangle effects of different dietary molecules on hormone secretion and to unravel ligand-receptor relationships (54, 55). Finally, ASC-derived organoids have recently also been employed to study the complex interaction between the epithelium and enteric nervous system in co-culture experiments (56, 57).

Human ASC-derived organoids are more complex to grow than their murine counterparts and require additional activation of Wnt-signaling and inhibition of TGF-beta and p38 MAP kinase signaling to expand stem cells (58). This necessitates separate expansion and differentiation conditions, since Wnt activation blocks differentiation towards most intestinal lineages (58). Multiple differentiation strategies have been employed to generate human EECs in these organoids, such as withdrawing growth factors and inhibition of Notch and MEK signaling (40), or addition of short-chain fatty acids, including butyrate (59). Importantly, the differentiation of the human intestinal organoids is an endpoint process that exhausts the stem-cell pool and results in a limited lifespan of the resulting cultures of about a week (58). To circumvent this limitation, a recent study performed transcriptomic analyses on human intestinal niche ligands and their receptors occurring in the stem-cell zone. The goal was to remove factors from the culture medium that would prevent differentiation and replace them with alternative signaling molecules that could maintain stem cells without interfering with downstream lineage specification (60). Based on their findings, the authors withdrew the p38 inhibitor and added the niche factors IGF-1 and FGF-2 (60). The resulting organoids expanded indefinitely and contained stem cells and most differentiated cell types, including EECs. As this work only assessed these conditions for the human ileum, future studies will have to show whether they are applicable to other parts of the small intestine or colon.

### Induced pluripotent stem cell-derived organoids

In analogy with ASCs, organoids can be derived from embryonic stem (ES) cells or their synthetic counterparts, induced pluripotent stem cells (iPS) cells. To do so, researchers mimic the developmental processes that shape maturing tissues in a dish (61). Endoderm is first specified by treating mouse or human pluripotent stem cells (PSCs) with Activin A, after which the mid/hindgut fate is induced by supplying FGF4 and WNT3A (62). (Fig. 3) The resulting spheroid cultures are incubated for 1 month in the same culture conditions established for mouse small intestinal organoids derived from ASCs (16, 62). During this period, the organoids mature into a columnar epithelium that develops villus-like structures, along with all mature cell types including 1% of the cells being EECs. Subepithelial fibroblasts and smooth muscle cells surround the human intestinal epithelial organoids and are likely derived from a small fraction of mesodermal cells induced after Activin A treatment (62). A recent study introduced functional enteric nervous system by co-culturing neural crest cells with developing human intestinal organoids, both derived from PSCs (63). Upon transplantation of these structures, functional networks of smooth muscle layers and neurons developed that were capable of concerted contraction and relaxation activities (63). Although EECs were often found associated with neuronal fibers, physical connection between the two were, in contrast to primary tissue, never found (63). This suggests a lack of maturity of one of the two players, or that these interactions require unknown exogenous factors or specific tissue architecture that does not arise spontaneously in the artificial environment.

PSC-derived intestinal organoids can be forced to differentiate towards EECs with the transient overexpression of Neurog3 (62, 64). These EECs have a wide range of distinct hormonal profiles and can respond to nutrient stimuli. In contrast to PSC-derived organoids, ASC-derived organoids have a strict regional identity based on the biopsy location, including the representation of EEC subtypes and hormones of that part of the gut (26, 49). The possibilities to induce regional specificity in PSC-derived cultures is still very limited. The original protocol to generate human PSC-derived organoids generally induced proximal small intestine hormones, with the notable exception of CCK, which was not produced (64). A short pulse of BMP activation in developing human gut tubes could specify PSC-derived cultures towards a colon-like fate that showed production of the typical large intestinal hormone *INSL5* in EECs (65). However, the ileal EEC repertoire could not yet be generated through these protocols, and might potentially be achieved by more subtle manipulation of BMP signaling during specification of the different parts of the gut.

The PSC-derived model is particularly interesting for disorders that have an origin during embryonic development, and that involve nonepithelial cell types. For example, PSC-derived enteric neurons have been successfully co-cultured with PSC-derived organoids and the formation of a fetal neuronal network between neurons and EECs has been observed (63). This is a promising approach to study developmental diseases of the enteric nervous system, such as Hirschsprung’s disease. Similarly, a recent study modeled different mutations in *NEUROG3* that cause a rare inherited EEC deficiency in humans using PSC-derived cultures and showed how these affect the stability and DNA-binding activity of the transcription factor (66).

The ability of PSC-based models to capture the embryonic development of a tissues makes these systems exquisitely suitable for the study of disorders that arise in development. The added ability to generate nonepithelial cell types together with intestinal epithelium allows assessing the cooperation and function of different cell populations. ASC-derived organoids, on the contrary, are simpler to handle and reliably generate differentiated cells that closely resemble those found in mature tissues. In fact, detailed transcriptomic comparisons have evidenced that ASC organoid EECs are virtually identical to EECs derived from primary tissue (32), which makes these organoids ideally suited to study the function and homeostatic turnover of EECs.

## Conclusions and Outlook

New models to study EECs, including intestinal organoids, have greatly increased insights into the developmental principles of these rare yet important sensory cells. The novel concept of hormonal plasticity demonstrates the surprising adaptability of the intestinal endocrine system. It reconciles observations of multihormonal cells with lineage-tracing data and greatly simplifies the EE lineage tree: there are only 5 different EEC lineages that produce mature cells with gradually changing hormonal profiles. These switches are imposed by increasing BMP signals during their journey along the crypt-villus axis. Blocking BMP signals or the migration process in the intestine would be a new strategy to increase numbers of GLP-1 producing cells and thus to shape the enteroendocrine landscape for diabetes treatment.

It is currently unknown why hormone production differs along the crypt-villus axis or along the gastrointestinal tract. Potentially, this is related to the function of the hormone or the sensory input it should respond to. For example, GLP-2 is a hormone associated with intestinal proliferation, a function required in the crypt (67). Villus-restricted and proximal intestine-enriched secretin is released in response to low pH (68). It is possible that luminal pH changes in the proximal small intestine are better sensed in the villus, and are buffered in the crypts. Differing pH along the villus epithelium has indeed been observed in the proximal but not distal small intestine (69).

Although the transcriptional effectors that drive the specification of individual EEC subtypes have been mapped, the signals upstream of these transcription factors remain elusive. Similarly, it is unknown how the stable differences in EEC subtype ratio arise along the gastrointestinal tract. DNA methylation patterns are strong candidates, but their influence remains to be functionally proven. Indeed, the ability to influence the regional identity of EECs would have great therapeutic value. Bariatric surgery, a common procedure that bypasses part of the proximal small intestine, is believed to be successful for glucose management due to rapid delivery of nutrients to the distal small intestine (70). Direct contact of distal EECs with these nutrients is believed to release a higher proportion of distal hormones. GLP-1 is more abundant in the distal small intestine, and is accordingly increased after bariatric surgery (70). GLP-1 acts then on beta cells in the pancreas and potentiates their insulin release in response to glucose, which gives patients better blood sugar control. Inducing a distal small intestine hormone signature in the proximal small intestine is expected to have a similar therapeutic outcome. GLP-1 is already the subject of multiple successful diabetes treatment, either by preventing its degradation or treating patients with GLP-1 receptor agonists (71). Other EEC hormones could be harnessed to target a plethora of human diseases (reviewed in (72)).

Finally, the availability of new human organoid systems gives researchers the opportunity to functionally test, in a human system, concepts that arose decades ago from studies in the mouse. This is necessary, due to certain key differences between the species. Mice and humans have different diets, and even produce different hormones in EECs, such as the human hormone motilin, which controls smooth muscle contractions of the upper gastrointestinal tract. It is therefore not unlikely that mouse and human EECs generate different hormonal outputs in response to the same nutrients or react to completely different signals. The use of human organoids to identify therapeutic secretagogues will ensure that identified molecules will have the desired effect in patients. However, the system is not limited to testing biological molecules and compounds but also compatible with more complex co-culture experiments. Enteric neurons and the microbiome are just 2 examples of a wide range of interactors that can be assessed in vitro for their ability to regulate gut hormones (56, 73, 74). The wealth of new single-cell data and availability of new human organoid tools has already overcome previous boundaries imposed by limited understanding of EEC fate specification and species differences. The next challenge for enteroendocrine biology will be to translate our knowledge to new therapeutic approaches that reshape the endocrine landscape in patient intestines to treat metabolic syndrome, diabetes, and potentially even inflammatory bowel diseases.

## Abbreviations

3D: three-dimensional
5-HT: serotonin
ASC: adult stem cell
CBC: crypt base columnar
CCK: cholecystokinin
EC: enterochromaffin cell
EEC: enteroendocrine cell
EGF: epidermal growth factor
GHRL: ghrelin
GIP: gastric inhibitory peptide
GLP-1: glucagon-like peptide-1
Neurog3: neurogenin-3
NTS: neurotensin
PSC: pluripotent stem cell
PYY: peptide YY

## Additional Information

***Disclosure Summary:*** JB and HG have nothing to disclose. HC is inventor on several patents related to organoid technology and holds the following additional positions: Both authors have submitted the ICMJE Form for Disclosure of Potential Conflicts of Interest. Conflicts that the editors consider relevant to the content of the manuscript have been disclosed.

***Unsalaried Advisory Positions:*** Clevers’s research group has spun out a nonprofit CRO organization, HUB, with roughly 45 employees. Hans Clevers is the (unpaid) Chief Scientific Officer of HUB. He has also filled rotating positions in international “Learned Societies” (e.g., ISSCR, AACR), Award Juries (e.g., Shaw Prize, Hong Kong; Breakthrough Prize, San Francisco) and in Scientific Advisory Boards (SABs) of various academic institutions (e.g., Crick Institute [London] and IMP [Vienna]).

***Salaried Advisory Positions:*** Hans Clevers is a co-founder and SAB member of Surrozen, a start-up in Silicon Valley, and an SAB member of Kallyope (New York), Merus (Utrecht) and Decibel (Boston). He is nonexecutive board member of Roche (Basel) and SAB member of the Roche subsidiary Genentech (San Francisco) since 2019. Hans Clevers is a scientific advisor for and investor in Life Sciences Partners, a biotech venture capital firm located in Amsterdam.

***Data Availability:*** Data sharing is not applicable to this article as no datasets were generated or analyzed during the current study.
